# Swimming training attenuates oxidative damage and increases enzymatic but not non-enzymatic antioxidant defenses in the rat brain

**DOI:** 10.1590/1414-431X20165310

**Published:** 2016-09-29

**Authors:** L.F. Nonato, E. Rocha-Vieira, R. Tossige-Gomes, A.A. Soares, B.A. Soares, D.A. Freitas, M.X. Oliveira, V.A. Mendonça, A.C. Lacerda, A.R. Massensini, H.R. Leite

**Affiliations:** 1 Laboratório de Inflamação e Metabolismo, Programa Multicêntrico de Pós-graduação em Ciências Fisiológicas, Sociedade Brasileira de Fisiologia, Centro Integrado de Pós-Graduação e Pesquisa em Saúde, Campus JK, Universidade Federal dos Vales do Jequitinhonha e Mucuri, Alto da Jacuba, MG, Brasil; 2 Laboratório de Biologia do Exercício, Programa Multicêntrico de Pós-graduação em Ciências Fisiológicas, Sociedade Brasileira de Fisiologia Centro Integrado de Pós-Graduação e Pesquisa em Saúde, Campus JK, Universidade Federal dos Vales do Jequitinhonha e Mucuri, Alto da Jacuba, MG, Brasil; 3 Núcleo de Neurociências, Departamento de Fisiologia e Biofísica, Instituto de Ciências Biológicas, Universidade Federal de Minas Gerais, Belo Horizonte, MG, Brasil

**Keywords:** Exercise therapy, Oxidative stress, Rat, Lipid peroxidation, Superoxide dismutase, Swimming training

## Abstract

Although it is well known that physical training ameliorates brain oxidative function after injuries by enhancing the levels of neurotrophic factors and oxidative status, there is little evidence addressing the influence of exercise training itself on brain oxidative damage and data is conflicting. This study investigated the effect of well-established swimming training protocol on lipid peroxidation and components of antioxidant system in the rat brain. Male Wistar rats were randomized into trained (5 days/week, 8 weeks, 30 min; n=8) and non-trained (n=7) groups. Forty-eight hours after the last session of exercise, animals were euthanized and the brain was collected for oxidative stress analysis. Swimming training decreased thiobarbituric acid reactive substances (TBARS) levels (P<0.05) and increased the activity of the antioxidant enzyme superoxide dismutase (SOD) (P<0.05) with no effect on brain non-enzymatic total antioxidant capacity, estimated by FRAP (ferric-reducing antioxidant power) assay (P>0.05). Moreover, the swimming training promoted metabolic adaptations, such as increased maximal workload capacity (P<0.05) and maintenance of body weight. In this context, the reduced TBARS content and increased SOD antioxidant activity induced by 8 weeks of swimming training are key factors in promoting brain resistance. In conclusion, swimming training attenuated oxidative damage and increased enzymatic antioxidant but not non-enzymatic status in the rat brain.

## Introduction

Physical training impacts several body systems including cardiovascular, musculoskeletal and nervous system (1). Studies have showed that regular exercise improves brain health and prevents or improves recovery from brain injuries (1) by enhancing neurotrophic factor levels (2) and modulating oxidative status (1).

Despite the fact that regular physical exercise is beneficial, it is well known that it can increase reactive oxygen species (ROS) production in different areas of the brain, as it consumes a higher amount of oxygen per unit of tissue mass, and contains high levels of peroxidizable lipids and excitotoxic amino acids, but low levels of antioxidants (3). Thus, depending on the intensity or duration of the physical training, it can be harmful, leading to oxidative stress and cell death (4).

Recently, our research group demonstrated higher brain resistance to brain injuries in animals submitted previously to 8 weeks of swimming training. Thus, mice exposed to experimental autoimmune encephalomyelitis showed both reduced pro-inflammatory cytokine levels and increased neurotrophic factor levels, as well as reduced oxidative damage, glutamate excitotoxity, necrosis and apoptosis in an oxygen-glucose deprivation model of cerebral stroke (1,5,6).

Although it is well known that prior exercise training ameliorates brain oxidative stress after injuries (1), there is little evidence addressing the influence of exercise training itself on brain oxidative stress and the findings are conflicting, due to different models (treadmill, voluntary or swimming) and different protocols of exercise (duration and intensity) used (7). The aim of this study was to investigate the effect of well-established swimming training on brain lipid peroxidation and antioxidant systems (enzymatic and non-enzymatic). We hypothesized that swimming training is able to decrease oxidative stress and increase brain antioxidant defenses.

## Material and Methods

### Animals

Male Wistar rats (n=15; 150–200 g) supplied by the Centro de Bioterismo da Universidade Federal de Minas Gerais (CEBIO UFMG vivarium) were housed under controlled environmental conditions (temperature 22±1°C and humidity 40–50%) with a 12:12-h light-dark cycle and free access to food and water. All experiments were conducted under the Protocol #139/2009 and approved by the university's Ethics Committee for Animal Experimentation (CETEA–UFMG). Efforts were made to avoid any unnecessary distress to the animals and ensure that the number of animals used was as low as possible. The CETEA directives are in compliance with NIH guidelines for the care and use of animals in research.

### Swimming exercise protocol

The swimming apparatus consisted of four independent glass pools with dimensions of 20×20×70 cm, with a closed loop of circulating water maintained at 32±1°C. Animals were placed in and removed from the apparatus using a fish net. Before the training protocol, all animals (n=15) were submitted to an adaptation protocol consisting of four daily swimming episodes of different durations (10, 15, 20, and 25 min). On the fifth day, all animals were subjected to a progressive load test to determine the maximal workload capacity (MWC). The MWC was determined by placing each animal in the water while progressively attaching to the tail a weight corresponding to 1% of its body weight every 3 min, until exhaustion (10 s of continuous submersion).

The exercise intensity of the endurance training was set at 60% of MWC. At this point, the animals were randomized into two groups: i) trained (TRA; n=8), which underwent swimming training for 30 min/day, 5 days/week, over 8 weeks (between 8:00 to 11:00 am) in individual tanks with 50 cm deep water, and ii) non-trained group (n=7), in which rats were placed on an acrylic platform in individual tanks with shallow water (5 cm) that did not permit swimming. One additional MWC test was conducted at the fourth week of exercise training. The rats were weighed weekly and the load for the endurance training was adjusted according to the most recent MWC for each rat (1,6).

### Tissue preparation

Forty-eight hours after the last session of exercise (fed state) the rats were rapidly killed by decapitation. The whole brain was immediately (<1 min) removed and washed in cold (4°C) modified Krebs-Henseilt preincubation solution containing 120 mM NaCl, 2 mM KCl, 0.5 mM CaCl_2_, 26 mM NaHCO_3_, 10 mM MgSO_4_, 1.18 mM KH_2_PO_4_, 11 mM glucose, and 200 mM sucrose. The brain samples were stored in a freezer at −80°C. At the day of the oxidative stress assays, the samples were homogenized in PBS at 10 g/100 mL (w/v) containing 140 mM KCl, pH 7.4, and centrifuged at 750 *g* for 10 min at 4°C, and the supernatant was then collected for the following assays.

### Thiobarbituric acid reactive substances (TBARS)

Lipid peroxidation was quantified by measuring the accumulation of TBARS in the homogenates and expressed as malondialdehyde (MDA) content. The content of MDA was measured at 532 nm (UV/visible U-200L spectrophotometer, Spectra Max 190, Molecular Devices, USA) using the method described by Ohkawa et al. (8). The results are reported as nmol of MDA/mg protein.

### Superoxide dismutase (SOD) assay

The activity of SOD (EC 1.15.1.1) was evaluated using a spectrophotometric method described by Srivastava et al. (9). The brain homogenate (0.05 mL) was incubated in a solution containing 50 mM KH_2_PO_4_, 1 mM diethylenetriaminepentaacetic acid (DTPA), and 50 mM EDTA, pH 7.4. The reaction was initiated by the addition of 0.2 mM pyrogallol. The oxidation of pyrogallol was measured at 420 nm (UV/visible U-200L Spectrophotometer) for 4 min at intervals of 10 s. A level of 50% inhibition was defined as 1 unit (U) of SOD, and the results are reported as U/mg protein.

### Total antioxidant capacity: the ferric-reducing antioxidant power assay (FRAP)

Non-enzymatic total antioxidant capacity (TAC) of brain samples was estimated by the ferric-reducing antioxidant power (FRAP) assay according to Benzie and Strain (10). The reduction capacity of the complex ferric Fe3+-TPTZ (ferric-tripyridyl triazine) to ferrous form Fe2+-TPTZ (ferrous-tripyridyl triazine) of antioxidants in acidic pH defines the brain’s antioxidant power. To measure non-enzymatic TAC, 30 μL of brain homogenate was incubated in a solution of 25 mL of sodium acetate buffer (0.3 M, pH 3.6), 2.5 mL TPTZ (10 mM) and 2.5 mL of FeCl_3_.H_2_O (20 mM) and 42 μL of deionized water. This mixture was homogenized and incubated in the dark at 37°C for 30 min. After cooling for 10 min, the samples were analyzed in duplicate in a microplate reader (SpectranMax^¯^190, Molecular Devices, USA) at 593 nm. Brain non-enzymatic TAC was expressed as millimolar of FeII equivalents determined from the standard curve of known concentrations of FeSO_4_ (0.0156 to 0.375 mM) and normalized by the amount of protein in the sample (FeII equivalent mM/µg protein).

### Protein assay

Protein content was measured according to Bradford's method (11) using bovine serum albumin (1 mg/mL) as the standard.

### Data analysis

Data are reported as means±SE. Statistical analyses were made using Student's *t*-test and Pearson's correlation. P<0.05 was considered to be statistically significant.

## Results

**Figure 1:**
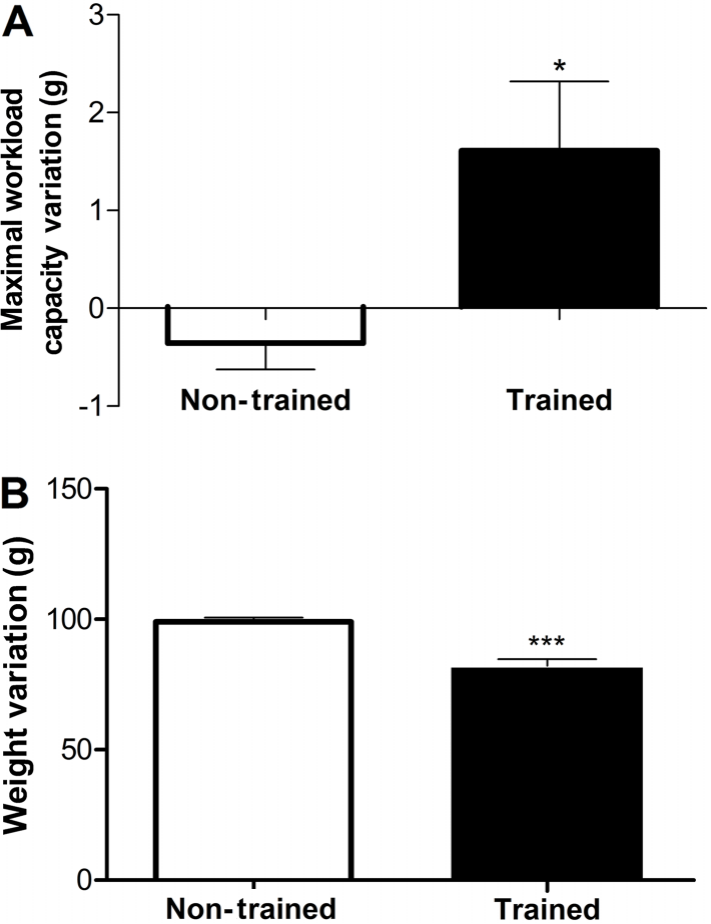
Effect of swimming training on maximal workload capacity and body weight variation. Data are reported as means±SE for n=15. *P<005, ***P<0.0001 compared with non-trained group (Student’s *t*-test).

In the present investigation, a significant variation (final – initial MWC) was found between non-trained (−0.30±0.2 g) and trained (1.6±0.7 g) groups in MWC (P<0.05: Figure 1A). Moreover, in spite of not finding a difference in body weight between sedentary and trained rats before starting the experiment (288.4±15.4 *vs* 260.0±36.0 g), a significant variation (final – initial weight) in total body weight in non-trained (99.0±1.6 g) *vs* trained (82.1±2.6 g) groups after the 8 weeks of swimming training was found (P<0.0001; Figure 1B).

**Figure 2:**
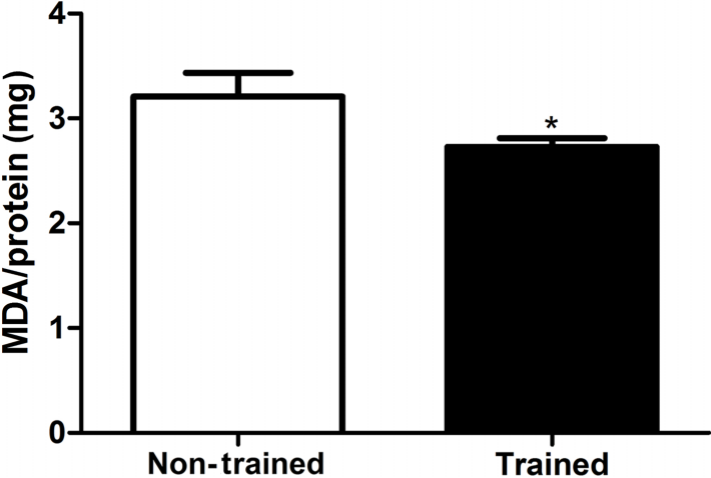
Effect of 8 weeks of swimming training on thiobarbituric acid reactive substances, measured by malondialdehyde (MDA) content in brain tissue. Data are reported as means±SE for n=13. *P<0.05 compared with non-trained group (Student’s *t*-test).

The effects of exercise training on TBARS content was measured (Figure 2). A difference between the non-trained group (3.2±0.2 MDA/mg protein) and the trained one (2.7±0.0 MDA/mg protein) was observed (P<0.05). Additionally, the basal data (sedentary values) are in agreement with other studies in the literature for TBARS content (7).

**Figure 3:**
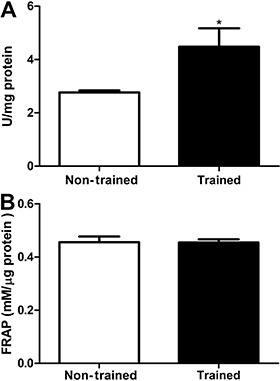
Effect of 8 weeks of swimming training on superoxide dismutase activity (*A*), and total antioxidant capacity of brain samples estimated by ferric-reducing antioxidant power (FRAP) (*B*). Data are reported as means±SE for n=13. *P<0.05 compared with non-trained group (Student’s *t*-test).

Swimming training increased brain antioxidant status as shown by an increased SOD activity in the trained group (4.7±0.7 U/mg protein) compared with the non-trained group (2.7±0.0 U/mg protein) (P<0.05; Figure 3A). No difference was observed on brain TAC between sedentary (0.45±0.0 mM/µg protein) and trained animals (0.45±0.0 mM/µg protein) (P>0.05; Figure 3B). Moreover, the basal data (sedentary values) are in agreement with others studies in the literature for SOD activity (12) and FRAP assay (13).

The correlation between MWC and changes in MDA content and SOD activity did not show a statistical significance (P>0.05).

## Discussion

In the present study, 8 weeks of swimming training attenuated oxidative damage and increased enzymatic but not non-enzymatic antioxidant activity in the rat brain. Although metabolic brain adaptations were found, it has been observed that exercise enhances and protects brain function through other mechanisms, such as plasticity and immune function (14). Among the exercise-induced benefits, the authors highlighted the increase in growth factors that protects neurons, assists neuronal plasticity and induces learning and memory (2,14).

Initially, increases in maximal workload capacity and maintenance of body weight composition were found, suggesting metabolic and aerobic adaptations. In agreement with previous studies from our research group, the 8 weeks of swimming training enabled the induction of metabolic adaptations, such as increased muscle and hepatic glycogen and MWC, and maintained body weight during the training period (1,6).

In respect to brain damage, the swimming training protocol employed in this study was effective against brain TBARS production, indicating a reduced oxidative damage. However, it has been demonstrated that young and old rats exposed to a swimming regime of 60–90 min per day, 5 days a week, for 6 weeks, showed no alteration in brain TBARS content (15). Another study revealed that a swimming exercise program (60 min/day, 5 days/week for 6 weeks) did not modify TBARS levels evaluated in cortical slices (16). In this context, the conflicting findings could be due to the different kinds and intensities of exercise training protocols applied, as well as distinct evaluated brain areas. Thus, we evaluated the brain as a whole, which certainly reflects better the physiological alterations triggered on brain by training. Moreover, a TBARS reduction was seen after 8 weeks, instead of 6 weeks, which results in a decreasing brain damage, reinforcing the time-dependent effect of exercise in promoting a brain-adaptive response.

We strongly agree with TBARS reduction being an attempt of the body to return to homeostasis, since exercise leads to ROS production (3), which in turn, could enhance antioxidant defenses in the brain. For this reason, we evaluated antioxidants enzymatic and non-enzymatic defenses to test this hypothesis.

No difference was found in brain non-enzymatic TAC between trained and non-trained rats. This suggests that swimming training does not enhance brain non-enzymatic total antioxidant capacity evaluated by the FRAP assay. No other study has investigated the effect of swimming training on brain non-enzymatic TAC. Other studies have reported no effect of swimming training on liver and skeletal muscle TAC, evaluated by the 2,2-diphenyl-1-picrylhydrazyl (DPPH) decomposition method (17). According to Benzie and Strain (10), ascorbic acid, α-tocopherol, proteins, and bilirubin are the main contributors to TAC of plasma, measured by the FRAP assay. The data suggest that swimming training does not modify the concentration of these antioxidants molecules in the brain.

On the other hand, the results showed that rats submitted to 8 weeks of swimming training had higher brain SOD activity. SOD activity, the first defense against ROS, converts two molecules of superoxide anion into a molecule of hydrogen peroxide and of oxygen, minimizing the formation of hazardous free radicals and lipid peroxidation (3). Souza et al. (16) demonstrated that rats submitted to 6 weeks of swimming training (60 min/day and 5 days/week), showed higher SOD activity in cortical slices than sedentary rats. Our results are in agreement with the reduced TBARS content observed in that study.

The brain is believed to be particularly vulnerable to oxidative stress because it contains high concentrations of polyunsaturated fatty acids (which are more susceptible to lipid peroxidation), consumes relatively large amounts of oxygen for energy production, and has lower antioxidant defenses compared to other organs (18). Thus, the influence of physical training on the central nervous system seems to be more notable in SOD activity than in MDA content. Moreover, SOD activity seems to be more sensitive to exercise-induced effects as it increases its activity to contain the excessive amounts of oxygen generated under physiological conditions, as well as in response to physical exercise (12). Radak et al. (2) showed a significant increase in SOD activity (in *corpus striatum* and brainstem) after 7.5 weeks of treadmill exercise (130% higher than the sedentary control). On the other hand, other studies showing the influence of physical exercise on TBARS content failed to reveal any significant difference (16) or revealed a modest TBARS reduction in trained animals (19).

We have previously demonstrated that 8 weeks of swimming training promotes neuroprotection through several adaptive mechanisms in response to chronic free radicals exposure (5). Therefore, we strongly agree that the reduced lipid peroxidation and increased SOD antioxidant activity are key factors in promoting brain health, once ROS and the associated oxidative damage have been pointed out as one of the possible regulating factors promoting brain resistance (15). Additionally, it is already known that exercise training can increase neurotrophic factors, such as brain-derived neurotrophic factor (BDNF) (14). BDNF protects against free-radical-mediated excitotoxicity injury by increasing SOD activity (20), which in turn, decreases oxidative damage (1) and pro-inflammatory cytokines (19), leading to neuroprotection (1,6). However, the link between neuroinflammation and oxidative stress needs to be better elucidated.

This study demonstrates that swimming training for 8 weeks can reduce brain lipid peroxidation, increase SOD activity, but not alter total antioxidant capacity in the rat brain. The results pointed out here are limited but raise some important questions for advancing scientific knowledge. In conclusion, it is reasonable to hypothesize that neuroprotection induced by swimming training involves antioxidant enzymes, but not non-enzymatic antioxidant defenses.
